# *In-vitro* Antitumor Activity and Antifungal Activity of Pennogenin Steroidal Saponins from* paris Polyphylla *var. *yunnanensis*

**Published:** 2011

**Authors:** Liancai Zhu, Jun Tan, Bochu Wang, Luhan Guan, Yuping Liu, Chao Zheng

**Affiliations:** a *Key Laboratory of Biorheological Science and Technology (Chongqing University), Ministry of Education, Bioengineering college, Chongqing University, Chongqing 400044, China.*; b *Department of Life Science & Chemistry, Chongqing Education College, Chongqing, 400067, China.*; c *Chemistry and Chemical Engineering College, Chongqing University, Chongqing, 400030, China.*

**Keywords:** *Paris polyphylla var. yunnanensis*, Pennogenin steroidal saponins, Antitumor activity, Antifungal activity

## Abstract

*Paris polyphylla Smith var. yunnanensis*, has been used in traditional Chinese medicine for its antibiotic and anti-inflammatory properties; in addition it has been used to cure liver cancer in particular. In this current study, *β*-ecdysterone (1) and three pennogenin steroidal saponins (2-4) were isolated from the EtOH extract of *Paris polyphylla var. yunnanensis*, and then tested for their antitumor and antifungal activities. Spectroscopic data was used to confirm their structures. Their antitumor properties were determined by using an MTT assay in addition to ethidium bromide and acridine orange staining techniques. Compounds 2, 3 and 4 exhibited significant anti-proliferation activities against HepG2 cells, with IC_50_ values of 13.5 μM, 9.7 μM and 11.6 μM respectively, obtained following 48 h treatment. Furthermore, we found these pennogenin steroidal saponins could induce HepG2 cells apoptosis at a concentration of 20 μM after 48 h treatment. Compounds 2, 3 and 4 were confirmed to exhibit moderate antifungal activity. The minimum inhibitory concentration (MIC) of compounds 2, 3 and 4 against *saccharomyces cerevisiae hansen* were 2.5 mg.mL^-1^, 0.6 mg.mL^-1^ and 0.6 mg.mL^-1^, respectively. The MIC of compounds 2, 3 and 4 against Candida albicans were 1.2 mgmL^-1^, 0.6 mg.mL^-1^ and 1.2 mg.mL^-1^, respectively. The analysis of the bioactivity-structure relationship shows that the sugar moiety plays a critical role in the activity of steroid moiety. Our results suggest that these three pennogenin steroidal saponins could be utilized to develop anticancer medicines.

## Introduction

Medical herbs have been used for a few thousands years and are considered to be one of the most promising sources of new medicines and leading compounds due to their therapeutic effects demonstrated in clinical use. *Paris polyphyllaSmith var. yunnanensis*, used as a Chinese traditional medicine, is widely distributed in China. It has been widely used due to its antibiotic and anti-inflammatory properties. In addition this herbal medicine has been used to treat liver cancer in particular (1). Certain steroidal saponins isolated from *paris polyphylla*have been reported to convey antitumor and immuno-stimulating properties (2-4). This paper reports the isolation, structure determination and biological activities of *β*–ecdysterone and three pennogenin steroidal saponins (2–4). We found that compounds 2-4 exhibited significant anti-proliferation activities against HepG2 cells and induced apoptosis of HepG2 cells. 

## Experimental

*Isolation*

The dried aerial part of *Paris polyphylla var. yunnanensis*, purchased from the Western Medicine City of Chongqing, China, were twice extracted with 10 volumes of EtOH under reflux at 8ºC for 60 min. The filtrate was combined and evaporated to achieve the ethanol extract. The ethanol extract was partitioned successively with chloroform, ethyl acetate and *n*-butanol. 

The *n*-butanol soluble fraction that showed major inhibitory activity on HepG2 cells, was subjected to fractionation using silica gel (Qingdao Ocean Chemical Company, China) and column eluted with CHCl_3_/MeOH (10 : 2-6 : 2 gradients) resulting in the formation of four fractions (F1-F4). F2 was then subjected to fractionation using silica gel column eluted with CHCl_3_/MeOH (12 : 2-10 : 2 gradients) to afford Compound 1. F3 was subjected to fractionation using Source 15 RPC (GE Healthcare Life Sciences) column eluted with MeOH/H_2_O (3 : 7-10 : 0 gradients) to afford compounds 2-4.

*Data of isolated compounds*

The spectra data of compounds 1-4 were measured: NMR on a 500 MHz Bruker DRX-500 instrument (Bruker, German) and MS on a VG Auto-spec3000 mass spectrometer (VG, England). 

*β–ecdysterone*

 White needle crystal; ESI-MS* m/ z *: 479 [M - H ] ^-^; ^1^H-NMR(500MHz, DMSO-d_6_) : δ_H_ 0.74 (3H, s, H-19), δ_H_ 0.82 (3H, s, H-18), δ_H_ 1.04 (3H, s, H-21), δ_H_ 1.06 (6H, s, H-26, H-27), δ_H_ 5.65 (1H, s, H-7), δ_H_ 3.13 (1H, d, J = 9.8Hz, H-22), δ_H_ 3.05 (1H, t, H-9), δ_H_ 4.39 (1H, m, H-2), δ_H_ 4.51 (1H, br s, H-3); ^13^C NMR (125 MHz, DMSO-d_6 _) Table 1. 

**Table 1:** The ^13^C-NMR chemical shifts of compounds 1-4 (DMSO-d_6_,δ).

**Aglycone moiety**	**Compound**				**Sugar moiety**	**Compound**		
	**1**	**2**	**3**	**4**		**2**	**3**	**4**
1	36.7	37.4	37.7	36.9		glc	glc	glc
2	66.9	30.2	30.9	30.9	1	98.7	98.2	100.1
3	66.7	78.2	77.9	78.1	2	76.9	76.6	78.0
4	31.0	38.1	37.7	37.7	3	76.4	75.8	75.4
5	50.2	140.8	140.3	140.3	4	71.0	76.2	76.3
6	203.0	121.8	121.4	121.4	5	76.6	76.0	76.0
7	120.6	31.3	31.3	31.3	6	63.4	63.0	63.3
8	165.5	31.8	31.4	31.4		rha	rha	rha
9	33.3	50.0	49.6	49.6	1	100.5	107.8	100.4
10	37.8	36.8	36.9	36.4	2	72.3	72.5	72.5
11	20.4	20.5	20.1	20.1	3	72.8	70.5	71.3
12	31.7	31.7	31.6	31.4	4	72.6	81.3	71.9
13	50.2	44.1	43.7	43.7	5	68.4	68.0	68.0
14	83.1	52.4	52.0	52.0	6	18.2	17.2	18.2
15	30.0	32.0	31.6	31.6			rha	ara
16	20.4	88.8	88.4	88.4	1		100.5	101.1
17	48.8	89.4	88.9	89.0	2		72.0	82.5
18	17.3	17.5	17.2	17.2	3		72.4	78.3
19	21.1	19.5	19.1	19.0	4		75.8	86.9
20	75.9	44.8	44.4	44.4	5		68.5	61.3
21	20.2	9.8	9.4	9.4	6		17.8	
22	75.9	109.2	108.8	108.8			rha	
23	24.0	30.6	30.9	30.9	1		100.2	
24	41.5	29.2	28.1	63.0	2		71.9	
25	75.8	29.5	29.1	29.1	3		70.5	
26	26.2	66.3	65.9	65.9	4		72.1	
27	29.1	17.0	16.8	16.8	5		68.5	
					6		16.8	

*pennogenin-3-O-α-L-rhamnopyranosyl (1→2)-β-D-glucopyranoside*

Colorless needle crystal; ESI-MS* m/ z *: 737 [M - H ] ^-^; ^1^H-NMR(500MHz, DMSO-d_6_): δ_H_ 0.68 (3H, d, J = 6.0 Hz, H-27), δ_H_ 0.98 (3H, s, H-18), δ_H_ 1.13 (3H, s, H-19), δ_H_ 1.23 (3H, d, J = 6.8 Hz, H-21), δ_H_ 1.86 (3H, d, J = 7.7 Hz , rha H-6′), δ_H_ 3.55 (2H, m, H-26), δ_H_ 3.82 (1H, m, H-3), δ_H_ 5.00 (1H, d, J = 4.5 Hz, glc H-1), δ_H_ 5.24 (1H, s, H-6), δ_H_ 6.37 (1H, s, rha H-1′); ^13^C NMR (125 MHz, DMSO-d_6 _) Table 1.

*pennogenin-3-O-α-L-rhamnopyranosyl (1→4)-α-L-rhamnopyranosyl*

*(1→4)-[α-L-rhamnopyranosyl (1→2)]-β-D-glucopyranoside*. 

Colorless needle crystal; ESI-MS* m/ z *: 1029 [M-H] ^-^;^ 1^H-NMR(500MHz, DMSO-d_6_): δ_H_ 0.69 (3H, d, J = 5.7 Hz, H-27), δ_H_ 0.94 (3H, s, H-18), δ_H_ 1.08 (3H, s, H-19), δ_H_ 1.22 (3H,d, J = 7.4 Hz, H-21), δ_H_ 1.58 (3H, d, J = 6.8 Hz, rha H-6′), δ_H_ 1.60 (3H, d, J = 6.9 Hz, rha H-6′′), δ_H_ 1.86 (3H, d, J = 7.7 Hz , rha H-6′′′), δ_H_ 3.60 (2H, m, H-26), δ_H_ 3.85 (1H, m, H-3), δ_H_ 4.98 (1H, d, J = 4.5 Hz, glc H-1), δ_H_ 5.24 (1H, s, H-6), δ_H_ 5.78(1H, s, rha H-1′), δ_H_ 6.27 (1H, s, rha H-1′′), δ_H_ 6.35 (1H, s, rha H-1′′′); ^13^C NMR (125 MHz, DMSO-d_6 _) Table 1.

*24-α-hydroxyl-pennogenin-3-O-α-L-rhamnopyranosyl (1→2)-[α-L-arabinofuranosyl (1→4)]-β-D-glucopyranoside*. Colorless needle crystal. ESI-MS *m/z* : 885 [M-H]^-^; ^1^H-NMR(500MHz, DMSO-d_6_): δ_H_ 0.70 (3H, d, J = 5.7 Hz, H-27), δ_H_ 0.94 (3H, s, H-18), δ_H_ 1.07 (3H, s, H-19), δ_H_ 1.22 (3H, d, J = 6.9 Hz, , H-21), δ_H_ 1.58 (3H, d, J = 6.0 Hz, rha H-6′), δ_H_ 3.52 (2H, m, H-26), δ_H_ 3.83 (1H, m, H-3), δ_H_ 4.98 (1H, d, J=4.5 Hz, glc H-1), δ_H_ 5.30 (1H, s, H-6), δ_H_ 5.82 (1H, s, ara H-1), δ_H_ 6.25 (1H, s, rha H-1); ^13^C NMR (125 MHz, DMSO-d_6 _) Table 1. 

*Assay for antitumor activity*

*Cell culture*

 HepG2 cells were cultured in RPMI 1640 medium (HyClone, USA) supplemented with 10% fetal bovine serum (HyClone, USA), 100 units/mL penicillin, 100 μg/mL streptomycin, L-glutamine (0.03%, w/v) and sodium bicarbonate (2.2%, w/v). The cell cultures were kept in a humidified incubator containing 5% CO_2_ set at 37°C. Subcultures were performed with 0.05% trypsin and 0.02% EDTA in phosphate-buffered saline solution (Gibco BRL Co., USA).

*MTT assay*

The MTT assay was performed according to the method set out by Mosmann(5). The HepG2 cells were plated into 96-well microtiter plates at a density of 1×10^4^ cells/well. After 24 h, the culture medium was replaced with 200 μL RPMI 1640 medium supplemented with 10% fetal bovine serum containing varying concentrations (0, 5, 10, 20, 40 and 80 μM) of compound 2, 3 and 4. The cells were then subjected to incubation for 24 and 48 h. The final concentration of solvent was less than 0.1% in the cell culture medium. The culture solutions were removed and replaced with 90 μL of culture medium. Ten microliters of sterile filtered MTT (Sigma, USA) solution (5 mg/mL) suspended in PBS (pH = 7.4) was added to each well to achieve a final concentration of 0.5 mg MTT/mL. The cells were then incubated at 37°C for 4 h. After the medium and unreacted dye was removed, 200 μL of DMSO was added to each well. The absorbance at 490 nm of the dissolved solution was measured using a Bio-Rad 680 microplate reader (BIORAD, USA).

The relative cell viability (%) of the control wells containing cell culture medium without the tested compound was calculated by dividing the absorbance of treated cells by that of the controls in each experiment. The IC_50_ was calculated as the tested compound concentration by means of SPSS statistical software, which inhibits the growth of 50% of cells in relation to non-treated control cells.

*AO/EB staining assay*

The cells were cultured on coverslips and kept in a 60 Petri dish for 24 h before treatment. Following treatment for 48 h with compounds 2, 3 and 4 at a concentration of 20μM, the cells without fixation were loaded with 100 μL of freshly-prepared AO/EB (Sigma, USA) staining solution (100 μg/mL). The cells were then immediately (less than 20 mins) observed under a fluorescence microscope (OLYMPUS, JAPAN).

*Assay for antifungal activity*

The antifungal activity against *saccharomyces cerevisiae hansen* and *candida albicans*of compounds 1-4 was evaluated by determining their minimum inhibitory concentrations (MIC), using broth microdilution techniques. The MIC values were determined in RPMI-1640 (Hyclone, USA). Stock solutions of pure compounds were twofold diluted with RPMI-1640 from 20.0 to 0.6 mg/mL and aliquoted into test tubes. Each tube was then inoculated with 25 μL of a standardized solution containing 10^6^ cfu/mL fungi suspended in sterile NaCl solution. After 24 h of incubation at 37°, the MICs were determined by using the optical density of the solutions. The lowest concentration of drug that inhibited all fungal growth was determined as being the MIC.

## Results and Discussion

*Isolation and identification*

**Figure 1:**
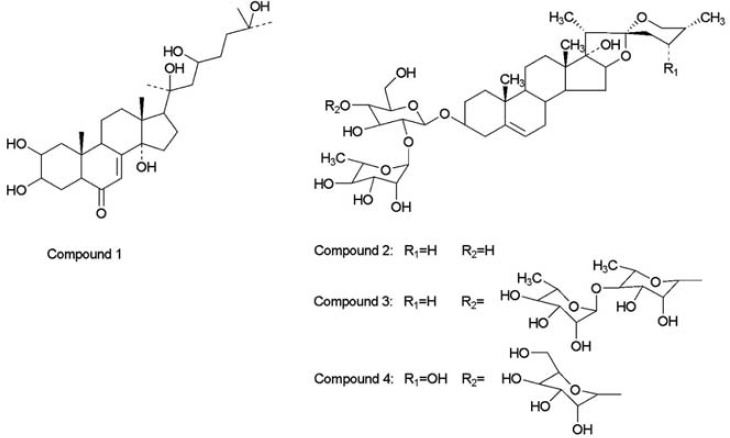
The structures of compound 1, 2, 3 and 4 from the EtOH extract of *paris polyphylla var. yunnanensis*

*The EtOH extract of *paris polyphyllavar. yunnanensis* was suspended in H*_2_*O and fractionated using chloroform, ethyl acetate and *n*-butanol, successively. The *n*-butanol soluble fraction, which showed major inhibitory activity on HepG2 cells, was repeatedly subjected to silica gel column chromatography and Source 15 RPC column chromatography to afford compounds 1-4. The structures of compounds 1-4 were analyzed using *^1^*H, *^13^*C NMR and MS. Finally, the structures were confirmed upon comparison with reference data. They were identified as *β*–ecdysterone(*1*) [6], pennogenin-3-O-*α*-L-rhamnopyranosyl (1→2)-*β*-D-glucopyranoside(*2*) [7], pennogenin-3-O-*α*-L-rhamnopyranosyl (1→4)-*α*-L-rhamnopyranosyl (1→4)-[*α*-L-rhamnopyranosyl (1→2)]-*β*-D-glucopyranoside(*3*) [7-8], and 24-*α*-hydroxyl-pennogenin-3-O-*α*-L-rhamnopyranosyl (1→2)-[*α*-L-arabinofuranosyl (1→4)]-*β*-D-glucopyranoside(*4*) [9], as shown in *Figure 1*. The last three are pennogenin steroidal saponins.*

*Antitumor activity*

Two experiments were carried out to determine the antitumor activity of the identified compounds. 

**Figure 2:**
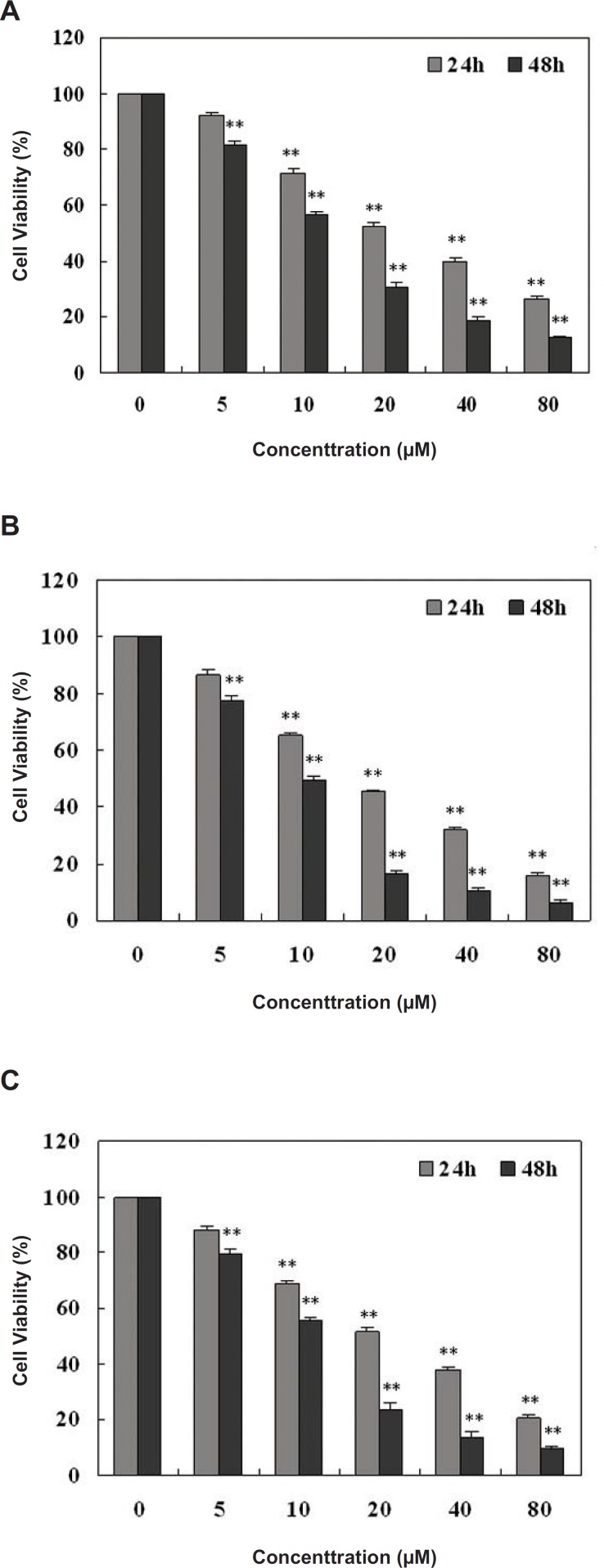
The effects of Compound 2, 3 and 4 on cell viability in HepG2 cells. (A) Compound 2; (B) Compound 3; and (C) Compound 4. The reported values are the means ± SD (n=5). **p<0.01 represents a significant difference between the experimental and control value.

The potential anti-proliferative effect of these identified compounds on the viability of HepG2 cells was evaluated by means of the MTT assay. Compound 1 showed no significant effect on inhibiting the growth and proliferation of HepG2 cells (data not shown). Cell viability is expressed as the mean percentage (±SD) of viable cells in comparison with untreated cells (taken as 100% viable) for different concentrations of compounds 2, 3 and 4. These compounds significantly inhibited the growth and proliferation of HepG2 cells in a dose- and time-dependent manner (Figure 2). 

The cell viability was shown to be 12.32%, 6.48% and 9.6% following treatment with 20 μM of compounds 2, 3 and 4 for 48 h, respectively. The estimated IC_50_ (50% of growth is inhibited) value of compounds 2, 3 and 4 obtained for 48 h treatment were 13.5 μM, 9.7 μM and 11.6 μM respectively, analyzed by SPSS software.

**Figure 3:**
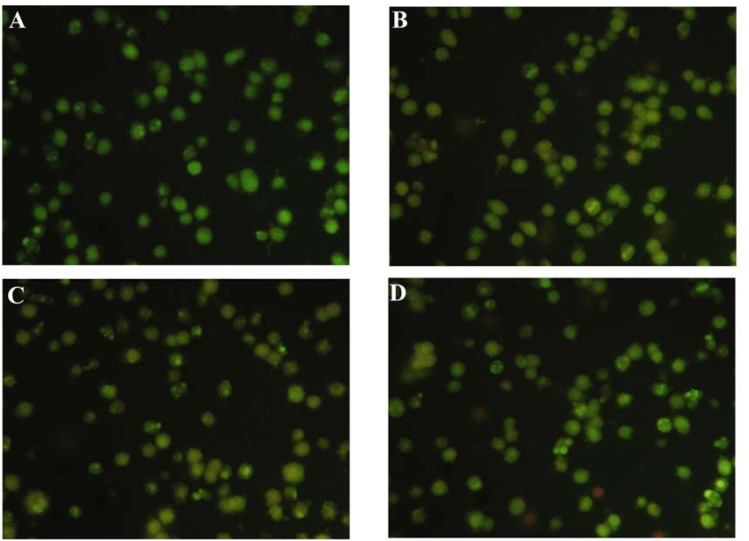
Apoptosis of HepG2 cells induced by compounds 2, 3 and 4 folowing 48 h treatment. After treatment with compounds 2, 3 and 4 and 48 h , the cells were stained with AO/EB at room temperature . The samples were observed under a fluorescence microscope immediately(×100) . (A) Control ; (B) 20 µM Compound 2 ; (C) 20 µM Compound 3;and (D) 20 µM Compound 4

To further address the cell death pattern, HepG2 cells after treatment for 48 h with compounds 2, 3 and 4 at a concentration of 20 μM were stained with the acridine orange and ethidium bromide (AO/EB), and immediately observed under a fluorescence microscope. AO/EB staining combines the differential uptake of fluorescent DNA binding dyes AO and EB and the morphologic aspect of chromatin condensation within the stained nucleus. This allows viable, apoptotic and necrotic cells to be distinguished from each another. Viable cells possess a uniformly bright green nucleus. Early apoptotic cells show bright green areas of condensed or fragmented chromatin within the nucleus. Necrotic cells exhibit a uniformly bright orange nucleus. After staining with AO/EB, the HepG2 cells showed a slight change in cell morphology with a very rough periphery. Effervescence, crumb-like structures, and nuclear fragmentation occurred, apoptotic bodies appeared, the permeability of cell membrane increased, and a window for both stained AO and EB was observed, which is a typical apoptosis characteristic (Figure 3). We believe that the apoptotic bodies visualized in our results are a specific effect caused by the activation of the cell apoptosis process within cancerous cells by treatment with either of the compounds and not just a mere overall toxic effect of the chemicals.

*Antifungal activity*

The antifungal test of the compounds from *paris polyphylla var. yunnanensis* gave the results listed in Table 2. 

**Table 2:** Antifungal activity of compounds 1, 2, 3 and 4

**Strains**	**MIC ( mg.mL** ^-1^ **)**			
	**Compound 1**	**Compound 2**	**Compound 3 **	**Compound 4**
*Saccharomyces cerevisiae hansen* *Candida albicans*	20.0 10.0	2.5 1.2	0.6 0.6	0.6 1.2

The results presented as minimal inhibitory concentration (MIC, mg.mL^-1^) show that compounds 2, 3 and 4 convey relatively higher antifungal activities against *saccharomyces cerevisiae hansen* and candida albicans than compound 1. The MIC of compounds 2, 3 and 4 against *saccharomyces cerevisiae hansen* are 2.5 mg.mL^-1^, 0.6 mg.mL^-1^ and 0.6 mg.mL^-1^, respectively. The MIC of compounds 2, 3 and 4 against *Candida albicans*are 1.2 mg.mL^-1^, 0.6 mg.mL^-1^ and 1.2 mg.mL^-1^, respectively. 

*Structure-activity relationships*

Compounds 2, 3 and 4, with the same steroidal saponin moiety, have different anticancer and antifungal activities. Compound 3 exhibits the strongest activity and also has the most sugar moieties among the three. This may suggest that there is a relation between the anticancer and antifungal activities of these compounds and the number of sugar moieties they contain. The water-solubility of these compounds increases in correlation with an increase in their sugar moiety number. Their anticancer and antifungal activity decreases in the order: compound 3 (4 sugar moieties) > compound 4 (3 sugar moieties) > compound 2 (2 sugar moieties). The hetero-sugar moiety causes the hetero-polarity of these compounds leading to different membrane permeability and selectivity, involved in the bioactivity of the compounds. The bioactivity-structure relationship of the compounds containing more sugar moieties than fourremains unclear. Too many sugar moieties may lead to an excessively large polarity, which would then impair the solubility of the active ingredient within the lipophillic medium of the biomembrane and thus block its transmembrane transfer. Sugar moiety has been reported to play a critical role in the activity of steroid moiety. Diosgenin, stigmasterol and solanidine, which have the same skeleton and different sugar moieties, show hetero-activity in the cell cycle arrest (10). OSW-1, characterized as a cholestane disaccharide, is an extraordinarily potent antitumor saponin. Whereas, the glycon OSW-1 and the disaccharide OSW-1 show no significant inhibition to the growth and proliferation of tumor cells (11). These facts all allude to the importance of a precise number of sugar moietiesin the bioactivity of saponins within living cells.

## Conclusion

In this work, *β*-ecdysterone (compound 1) and three other compounds with the same steroidal saponin moiety were isolated from *Paris polyphylla var. yunnanensis* and their structures were identified. We found that compounds 2, 3 and 4 not only inhibited cancer cell proliferation but in addition induced cancer cell apoptosis. These three pennogenin steroidal saponins may provide clues for designing a range of novel semi-synthetic and synthetic compounds as medicinal anti-cancer agents in the near future.
